# Charles Darwin's Reception in Germany and What Followed

**DOI:** 10.1371/journal.pbio.1000162

**Published:** 2009-07-28

**Authors:** Axel Meyer

**Affiliations:** Chair in Zoology and Evolutionary Biology, Department of Biology, University of Konstanz, Konstanz, Germany

## Abstract

150 years ago, Heinrich Bronn provided in the first German translation of Charles Darwin's *Origin of Species* a rather liberal interpretation, even adding his own view of Darwin's ideas in an additional 15th chapter. Ernst Haeckel widely popularized his view of Darwinian evolution based on his reading of this translation. This was long seen - probably incorrectly - as the intellectual root of social Darwinism in Germany.

In Germany, Charles Darwin's thinking was accepted very quickly after the publication of *On the Origin of Species* in November, 1859. This was due, in no small measure, to the fact that a translation by the noted German paleontologist Heinrich Georg Bronn appeared in April, 1860, only months after the original publication [1]. Bronn's own research led him to several insights that paralleled those of Darwin, resulting in a translation that was quite liberal and included the addition of numerous footnotes; but perhaps most importantly, Bronn added a new final chapter (chapter 15) to Darwin's book [2]. In these final notes, Bronn summarized his assessments of and conclusions on Darwin's *Origin* in 26 pages [2]. He outlined what he thought Darwin had meant to say, partly reinterpreted it, and critiqued it. Darwin welcomed this discussion, and 18 letters were exchanged between the two men. In subsequent editions of *Origin*, Darwin developed his theory further through such feedback. Bronn's critical epilogue was partly inspired by his adherence to an idealistic—even romantic—and teleological *Naturphilosophie* that viewed evolution as a progressive development toward perfection; this has at least been long thought, explaining why Bronn used the word, in both text and title of the translation, *vervollkommnet* (perfected) for Darwin's word “favored.” Bronn also freely translated Darwin's “struggle for existence” into *Kampf ums Dasein*, which might be best translated back into English as “fight for existence or life,” a phrase that Darwin himself was not entirely happy with. New interpretations of Bronn's work and his influence on Ernst Haeckel and evolutionary thought in Germany are presented in the new book by Sander Gliboff, *H. G. Bronn, Ernst Haeckel, and the Origins of German Darwinism: A Study in Translation and Transformation* [3].

Haeckel, who was the most influential don of German zoology for several decades, probably read Darwin's *Origin* in German during his PhD work in Jena, since his command of English was not particularly good. The main reason why all of this is of greater, even political, interest beyond issues in the history of science, is that Ernst Haeckel is widely seen—although this is disputed among historians of science—to be in an unholy intellectual line from Darwin to social Darwinism and eugenics in the early twentieth century, eventually leading to fascism in Nazi Germany. Creationist and intelligent-design advocates worldwide tirelessly perpetuate this purported but largely unsubstantiated connection between Darwin, Haeckel, and Hitler [4]. Such efforts are particularly and unnecessarily divisive in this “Darwin year,” when we celebrate not only the 150th anniversary of the publication of *Origin*, but also Darwin's 200th birthday. Furthermore, they do not do justice to Haeckel's understanding of Darwinian evolution by natural selection with all its unpredictability, but, more importantly, seem to aim to further undermine the acceptance of evolution by an often still surprisingly skeptical lay audience.

Haeckel was, by far, the most successful popularizer of science for more than a generation in Germany [5],[6]. His books were printed in large numbers, translated into several languages, and strongly influenced scientists and layman alike [7],[8]. Haeckel idealized Darwin. He dedicated his seminal work “Generelle Morphologie” to Darwin (as well as to his teacher Carl Gegenbaur, Johann Wolfgang von Goethe, and Jean-Baptiste de Lamarck) and visited him in Down House in August of 1866. When Darwin was presented with a copy of Haeckel's book, he received it with gracious thanks and remarked (in a letter to Haeckel from 18 August 1866), “You confer on my book, the *Origin of Species*, the most magnificent eulogium which it has ever received.” Haeckel misunderstood many aspects of Darwin's ideas, and perhaps his typical German quest for laws of nature was ill-founded. Nonetheless, he was instrumental in propagating the principle of evolution by natural selection to the then very influential community of German biologists that had long adhered to lamarckian ideas.

Bronn died in 1862, and later editions of Darwin's seminal book were translated into German by Julius Victor Carus. A son of Friedrich Emil Suchsland, a publisher of Darwin's translations, asked Darwin for permission for a new translation of later editions of the *Origin*, claiming that his theory had been widely misunderstood in Germany because of shortcomings in the early translation by Bronn. This interpretation of Bronn's skewed understanding of Darwin, and its effects on Haeckel's own misunderstanding, continued in the history of science until recently [9],[10].

Gliboff's new book [3] clarifies that, although Bronn never completely accepted Darwin's idea of species transformation, he did immediately recognize that Darwin's *Origin* was a huge advance toward a more comprehensive science of life, which Bronn himself had long sought to establish. Gliboff's book is a very readable, concise, and an important contribution that will help to rectify some entrenched misunderstandings about the history of evolutionary thinking in Germany. It needs to be said, however, that Gliboff almost summarily ignores a large body of work from German scholars on these aspects of the history of evolutionary biology. It was Thomas Junker and others [9],[10] who clarified the historical role of Bronn's translation of Darwin on the acceptance and perception of Darwinian evolution in German. Also, other researchers from Jena (where Haeckel spent 47 years of his career as professor), such as Uwe Hoßfeld, deserve more credit than Gliboff gives them [7],[11]. The omission of the insights of these scholars detracts from the impact that this book should have.

Gliboff makes an important contribution by pointing out that Bronn's liberal translation of Darwin altered the precise meanings of Victorian English words to fit a contemporaneous German sensibility. Much of this can be attributed to Bronn's interpretations of Darwin. Bronn had been thinking along lines perhaps parallel to Darwin's, aiming to modify Darwin where he thought he knew better than Darwin himself. Bronn and Haeckel initially had difficulty dealing with Darwin's theory because it described variation, diversity, and changes that did not seem to obey predictions from “natural laws”. However, their work—just like the insights of the following generations of evolutionary biologists—extended Darwin's theory beyond that which was known to Darwin himself and led to a healthy discourse among different interpretations. One should not forget that Darwin's thoughts were only the beginning of modern evolutionary biology. A huge amount of research in evolutionary biology in the 150 years since the publication of *Origin* added, extended, and modified Darwin's initial thinking—as should be expected in any vibrant scientific discipline—but did not contradict the core tenets of Darwin.[Fig pbio-1000162-g001]

**Figure pbio-1000162-g001:**
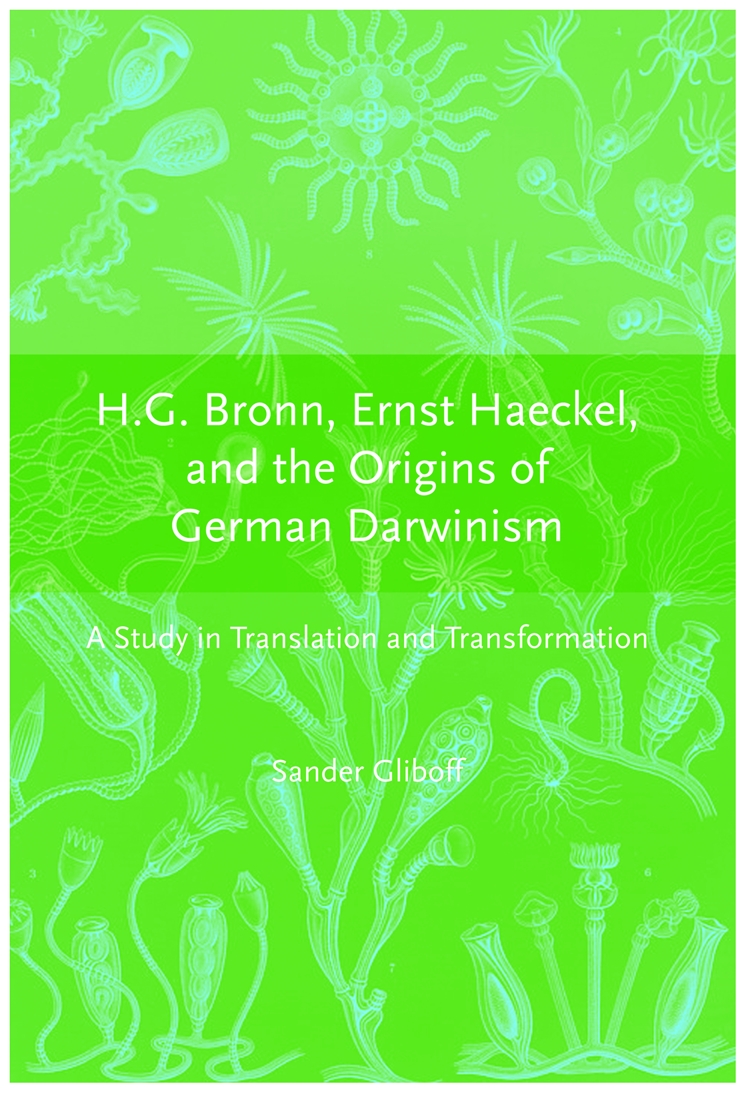
Gliboff S (2008) H.G. Bronn, Ernst Haeckel, and the origins of German Darwinism: a study in translation and transformation. Cambridge (Massachusetts): MIT Press. 259 p. ISBN (hardcover): 978-0-262-07293-9. US$35.00.
